# A prospective analysis of the effect of neighbourhood and individual social capital on changes in self-rated health of people with chronic illness

**DOI:** 10.1186/1471-2458-14-675

**Published:** 2014-07-03

**Authors:** Geeke Waverijn, Mary K Wolfe, Sigrid Mohnen, Mieke Rijken, Peter Spreeuwenberg, Peter Groenewegen

**Affiliations:** 1 Nivel, Netherlands Institute for Health Services Research, Otterstraat 118 – 124, 3513 CR Utrecht, The Netherlands; 2 At the time of this research, master student in Urban Geography, Utrecht University, Heidelberglaan 2, 3584 CS Utrecht, The Netherlands; 3 RIVM, National Institute for Public Health and the Environment, Antonie van Leeuwenhoeklaan 9, 3721 MA Bilthoven, The Netherlands; 4 Department of Sociology, Department of Human Geography, Utrecht University, Heidelberglaan 2, 3584 CS Utrecht, The Netherlands

**Keywords:** Social capital, Neighbourhood, Chronic illness, Self-rated health, Multilevel modelling

## Abstract

**Background:**

Social capital in the living environment, both on the individual and neighbourhood level, is positively associated with people’s self-rated health; however, prospective and longitudinal studies are rare, making causal conclusions difficult. To shed more light on the direction of the relationship between social capital and self-rated health, we investigated main and interaction effects of individual and neighbourhood social capital at baseline on changes in self-rated health of people with a somatic chronic disease.

**Methods:**

Individual social capital, self-rated health and other individual level variables were assessed among a nationwide sample of 1048 non-institutionalized people with a somatic chronic disease residing in 259 neighbourhoods in the Netherlands. The assessment of neighbourhood social capital was based on data from a nationwide survey among the general Dutch population. The association of social capital with changes in self-rated health was assessed by multilevel regression analysis.

**Results:**

Both individual social capital and neighbourhood social capital at baseline were significantly associated with changes in self-rated health over the time period of 2005 to 2008 while controlling for several disease characteristics, other individual level and neighbourhood level characteristics. No significant interactions were found between social capital on the individual and on the neighbourhood level.

**Conclusions:**

Higher levels of individual and neighbourhood social capital independently and positively affect changes in self-rated health of people with chronic illness. Although most of the variation in health is explained at the individual level, one’s social environment should be considered as a possible relevant influence on the health of the chronically ill.

## Background

Beyond characteristics of the individual, physical and social features of the environment are also related to variations in human health and disease [1-5]. This has sparked considerable interest in the association between neighbourhood context and health outcomes. An important neighbourhood characteristic is social capital. Social capital is rooted in social relations and grows through ties within networks, such as families, communities, or neighbourhoods. It works through shared norms and mutual trust, and it is a resource that is utilized by both individuals and groups. Social capital influences the behaviour of those within the social circle in which it operates. Unlike human capital, social capital cannot be seen as the 'possession' of an individual but rather as a supply of resources that exists within the structure of relations between actors [6]. People living in neighbourhoods with more social capital experience better health, independent of socio-demographic characteristics of the neighbourhood and physical characteristics of the residents [7-15].

Although there is evidence that neighbourhood social capital is an important contextual influence on health, there is little clarity about whether this influence differs for people who experience more severe health problems. In this article we study the relationship between social capital and health specifically for people with a chronic illness. We investigate change in self-rated health over time in relation to social capital in neighbourhoods at baseline. Moreover, we study both collective and individual social capital. Collective social capital is located on the geographical level and is based on the level of social capital of the inhabitants of a neighbourhood.

The increasing prevalence of chronic illness in the population [16] calls for more insight into the factors that contribute to health and wellbeing. Most research concerning the health of people with chronic illness neglects the contextual effect of the social environment. The residential environment might be especially important when disabilities potentially limit the action space of people with chronic illness. Social capital can be seen as a potential supportive resource that helps people cope with the consequences of their illness. It may also encourage them to engage in a lifestyle that prevents deterioration of health.

Current research on social capital and health is most often cross-sectional, which makes it difficult to establish the direction of the relationship between social capital and health. In most studies it cannot be ruled out that selection effects are responsible for the association between the neighbourhood environment and health. This means that we cannot exclude the possibility that people with better health are selected into certain neighbourhoods and that reverse causality might play a role, i.e. that good health is a determinant of social capital rather than the other way around. Neighbourhood characteristics (such as the level of deprivation, neighbourhood problems, and social capital) have been linked through prospective research to incidence of disease [17-19], functional disability and functional decline [20-22], prevalence of mental disorders and mental hospitalization [23,24] and mortality rates [19,25-27]. Murayama et al. [28] in their review of prospective multilevel studies on social capital and health identify one prospective study about the effect of area level social capital on self-rated health, which shows that high individual and area social trust have a positive effect on self-rated health [11]. A study published after this review examined the effect of neighbourhood social capital on health among a sample of pregnant women in Brazil [29]. With regard to the effect of social capital over time on the individual level it was found that frequent contact with neighbours is associated with better self-rated health over a period of two years [30,31]. It can be expected that people with chronic illness experience more pronounced health changes in shorter periods of time than the general population. Therefore, this population is especially suitable to study the effect of social capital (on both the individual and neighbourhood level) on changes in health.

Social capital has been conceptualized and measured both at the collective (e.g. neighbourhood) and individual level. Although there are mixed results on the strength of the association, both neighbourhood social capital [7-15] and individual social capital [32-34] are positively related to self-rated health. Social capital on the individual level exists in relations between specific actors [35], while social capital on a geographical level is a collective and non-exclusive good that can also benefit those who do not personally invest in the social structure they belong to [6]. As such, people with less individual social capital, who seldom participate or invest in their neighbourhood, can potentially benefit from living in a high social capital community, e.g. because this community is a safe and welcoming environment in which to stay physically active. This might be important for people with chronic illness who might be less able to participate in their neighbourhood (and gain individual social capital) because of the limitations their health condition presents. Individual responses to survey questions about relationships in the neighbourhood can be used to construct a measure of collective social capital [36] through aggregation of responses or econometric analysis [37].

Interactions between collective and individual social capital have been reported [38-41]. Results suggest that social capital on a geographical level does not uniformly benefit individuals in the same way. People with higher levels of trust and civic participation are more likely to report good health than people with low levels of trust and civic participation rates in countries with high levels of trust and civic participation rates. But this is not the case in countries with low levels of trust and civic participation rates. This means that in countries with high levels of social capital, people with high levels of individual social capital benefit more from this than people with a low level of individual social capital [41]. Furthermore, it was found that neighbourhood social capital can compensate for a lack of individual social capital [40]. An accumulating effect might also occur if social ties at the individual level strengthen the relationship between neighbourhood level social capital and health [38].

This research aims to contribute to existing literature by examining the relationship between social capital, both on the individual and on the neighbourhood level, and changes over time in self-rated health among people with a somatic chronic disease. We test the following hypotheses:

1. A higher level of individual or neighbourhood social capital exerts a positive influence on changes in the self-rated health of people with a medically diagnosed somatic chronic disease.

2. There is an interaction between the effect of neighbourhood social capital and individual social capital on changes in self-rated health.

## Methods

### Geographical unit

Neighbourhoods can be spatially defined based on key area codes [10], defined as quantitative administrative units [9] or more qualitatively designated by the individual perceptions that residents have of “the area around where [they] live and around [their] house” [15]. In our study neighbourhoods are spatially defined based on 4-digit postal codes. Postal codes in the Netherlands are used to identify relatively small geographical areas that comprise between 1–8 km^2^ with an average of 1800 households and 4000 residents per postal code area [42]. Due to differences in population density, neighbourhoods comprise relatively small areas in cities and larger areas in rural municipalities. In our study sample there are on average 4.0 chronically ill residents per neighbourhood (SD = 6.7), in 259 urban and rural postal code areas.

### Data

Three data sources were used to test our hypotheses. Two of these data sources contained information about the neighbourhood. The first dataset was the ‘Housing and living survey 2006’ (WoON) as previously used by Mohnen and colleagues [7]. This provided us with an independent measure of neighbourhood social capital, based on an average of 19 respondents per neighbourhood [43]. The WoON survey data were collected among a representative sample of the general population under the authority of the former Ministry of Housing, Spatial Planning, and Environment. The WoON data provide an independent measure of social capital, established separately from the perceptions of the study sample of people with chronic illness. In total, 64005 respondents participated in the WoON 2006 survey.

A second dataset containing neighbourhood control variables, based on aggregated register information, was provided by Statistics Netherlands and was added to these data [42].

The third dataset yields information on the individual level and was used to construct a measure of self-rated health and individual social capital. These data are based on longitudinal survey data from the ‘Nationaal Panel Chronisch zieken en Gehandicapten’ (National Panel of the Chronically ill and Disabled or NPCD). This is a nationwide prospective panel in The Netherlands established to gather information on the consequences of chronic disease and disability from a patient perspective [44]. For the NPCD, participants are recruited from random samples of general practices that are drawn from the Dutch Database of General Practices. For the present study, participants with a chronic disease were recruited from 44 different practices. They were selected using the following criteria: diagnosis of a somatic chronic disease by a certified medical practitioner, aged > 15 years, not permanently institutionalized, aware of the diagnosis, not terminally ill (life expectancy > 6 months according to their general practitioner), mentally capable of participating, and sufficient mastery of Dutch. Annually, 500 new panel members are selected via the standardized procedure to replace panel members who withdrew or who had participated for the maximum term of four years. All non-institutionalized people in The Netherlands are listed with a general practice and general practitioners keep lifelong patient files which are carried over in case a patient residentially relocates. NPCD is registered with the Dutch Data Protection Authority; all data are collected and handled in accordance with the privacy protection guidelines of this Authority. Panel members fill in questionnaires at home twice a year. The present study used panel data from 2005 to 2008. For the purpose of this study we confined our sample to respondents to the questionnaire of 2005 and from whom we had at least one additional assessment of self-rated health in later years as well as a baseline measure of individual social capital. In April 2005, a survey was sent to 2164 panel members diagnosed with a chronic disease, for whom we possessed neighbourhood social capital information. A total of 1914 people completed the questionnaire in 2005 (88%) and a total of 1048 completed an additional questionnaire in the following years and provided information to construct measures of self-rated health and individual social capital. Those who only filled out the questionnaire in 2005, and did not drop out before the next questionnaire because they had participated for a maximum of 4 years (this was the case for 29% of the people who participated in 2005) did not differ from those who did complete one of the next questionnaires in terms of age, education level, level of individual social capital, number of chronic illnesses, and the presence and severity of a disability. The people who filled out an additional questionnaire were a bit younger on average (57 versus 58 for those who did not participate in further measurements) and more often female (57% versus 52%), but these differences were not significant.

### Measurements

#### Health change

Self-rated health in 2005 was used as baseline health. The dependent variable was derived from one to three measurements of respondents’ self-rated health after 2005, as assessed with the scale ‘general health’ from the RAND-36 short-form health status survey [44]. In the RAND-36 survey respondents are asked to answer the following question: ‘In general how would you say your health is?’ with answers on a 5-point scale, ranging from ‘excellent’ to ‘poor’. Respondents then select answers that best describe how much they agree with four statements about their health on a 5-point scale ranging from ‘totally agree’ to ‘totally disagree’. The statements are: ‘I seem to get sick a little easier than other people’, ‘I am as healthy as anybody I know’, ‘I expect my health to get worse’ and ‘My health is excellent’. The scale score of perceived general health is the sum of these five items, rescaled to range from 0 (worst health) to 100 (best health) [45]. A higher score reflects better self-rated health. In our study, Cronbach’s alpha of the scale varied between 0.805 and 0.823 (depending on the measurement year), indicating a high internal consistency. This measurement instrument was found to have good discriminatory power among a sample of people with a chronic disease in Finland who were critically ill [46]. The correlation of this scale with objective health measures and physician-assessed health is high [47,48]. There are a maximum of four measurements of health for each respondent. All included respondents have the measurement of health at baseline in 2005 (independent variable). There are 959 respondents who have a measurement of health in 2006, 878 respondents who have a measurement of health in 2007, and 702 respondents who have a measurement of health in 2008. There are 625 respondents who have three additional measurements of health (after baseline measurement), 241 respondents who have two additional measurements of health, and 182 respondents who have one additional measurement of health. There are 790 respondents who have additional measurements of health in both 2006 and 2007, 638 respondents who have additional measurements of health in both 2006 and 2008, and 686 respondents who have additional measurements of health in 2007 and 2008. Because people participate in the NPCD for a maximum of four years, people who received their first questionnaire before 2005 or before 2004 were unable to participate after the measurement in 2007 and after the measurement of 2006 respectively.

#### Individual social capital

Individual social capital in the neighbourhood was derived from ten items from the NPCD questionnaire in 2005, which include: ‘I like the neighbourhood where I live’, ‘I feel connected to this neighbourhood’, ‘I know my neighbours well’ and ‘There is social interaction in this neighbourhood’. These questions are based on the items used to assess social capital in the neighbourhood in the ‘Housing needs survey 2002’ which was conducted under the authority of the former Ministry of Housing, Spatial Planning, and Environment. Respondents were asked to choose their level of agreement with these statements on a 5-point scale ranging from ‘totally disagree’ to ‘totally agree’. The scale score of individual social capital was a sum of these ten items, ranging from 10–50, with a higher score indicating higher levels of individual social capital in the neighbourhood. The reliability of this scale in our study was high (Cronbach’s alpha = 0.935).

#### Individual level control variables

Among the individual level variables are a number of demographic and illness characteristics, namely: sex, coded as a dummy variable; age, measured in years in 2005; educational attainment, coded as either low (no education until the lowest high school degree), average (vocational training and the highest two high school degrees), or high (university of applied sciences degree and university degree); net equivalent income in four categories (less than 900 euro, 900–1200 euro, 1200–1600 euro, more than 1600 euro). Net equivalent income is defined as the sum of the monthly net incomes (regardless of source) of all household members corrected for household composition [49]. We also included employment status (having a paid job or not) and marital status (being married or not). Three disease characteristics were included: the nature of the first diagnosed disease (including cardiovascular diseases, cancer, respiratory diseases, diabetes, musculoskeletal diseases, neurological diseases and digestive diseases); the number of chronic diseases (ranging from 1 to ‘3 or more’) as reported by the general practitioner; and self-reported severity of the disability (no disability or mild disability, moderate disability, severe disability), as assessed by a validated Dutch questionnaire containing questions about the ability to perform a number of activities in daily life [50]. Descriptive statistics of individual level variables are presented in Table 1.

**Table 1:** Descriptive statistics of individual variables of chronically ill (n = 1048)

	**Range**	**Mean**	**S.D.**	**Percent**
Health 2005		0-100	53.9	20.7
Health 2006		0-100	53.4	20.8
Health 2007		0-100	54.5	21.8
Health 2008		0-100	53.0	21.7
Change in health 2006–2008 vs. 2005		−55/60	11.4	14.9
Sex				
Male				43.3
Female				56.7
Age in years		16-95	56.9	15.0
Education				
Low				56.3
Middle				25.3
High				18.4
Income				
Less than 900				11.0
900-1200				21.2
1200-1600				18.9
More than 1600				16.6
Unknown				32.3
Index disease				
Cardiovascular disease				10.4
Cancer				2.8
Respiratory disease				42.6
Diabetes				11.9
Musculoskeletal disease				9.5
Neurological disease				5.5
Digestive disease				3.4
Unspecified other disease				13.9
Number of chronic diseases				
Number one				71.3
Two				20.3
Three or more				8.4
Severity of disability				
None or mild disability				70.3
Moderate disability				22.0
Severe disability				7.7
Marital status				
Unmarried				33.6
Married				66.5
Employment status				
Unemployed				58.6
Employed				40.0
Unknown				1.4
Individual level social capital		10-50	38.6	7.4

#### Neighbourhood social capital

Social capital is rooted within ties between individuals and through these ties resources are created. Therefore social capital is measured by specifically focusing on contacts between neighbours. ‘Neighbourhood social capital’ was assessed by five questions relating to contact among neighbours. Items address the following: contact with direct neighbours, contact with other neighbours, whether people in the neighbourhood know each other, whether neighbours are friendly to each other and whether there is a friendly and sociable atmosphere in the neighbourhood. Respondents were asked to choose their level of agreement with these statements on a 5-point scale ranging from ‘totally disagree’ to ‘totally agree’. An ecometric analysis was used to create the measure of neighbourhood social capital (as previously described by Mohnen and colleagues) [7]. The ecometric approach accounts for the dependency among the items that measure social capital, for different numbers of respondents in neighbourhoods and is adjusted for individual characteristics of the respondents. Neighbourhood level residuals are used to indicate neighbourhood social capital.

#### Neighbourhood control variables

Urbanity of the municipality and neighbourhood income were used as control variables at the neighbourhood level. The level of urbanity was based on a 5-point scale: rural, semi-rural, intermediate urban–rural, semi-urban, strongly urban. Income was assessed by the percentage of people in the highest income quintile in a neighbourhood. We aimed to control for ethnic diversity of a neighbourhood by including the percentage of immigrants, but as a consequence of its high correlation with the level of urbanity (correlation coefficient of 0.82), it was omitted from the analyses. Models run with ethnic diversity included instead of the level of urbanity did not differ significantly in the effect of our main outcome variables from models with the level of urbanity included instead of ethnic diversity. Table 2 shows the descriptive statistics of neighbourhood level variables.

**Table 2:** Descriptive statistics of neighbourhood characteristics (n = 259)

	**Data source**	**Year**	**Range**	**Mean**	**SD**
Urbanity of municipality	Stat. Neth^a^	2005	1-5	2.9	1.3
Percentage of population in the Highest income quintile	Stat. Neth	2005	1.9-28.5%	14.4	5.0
Percentage of immigrants	Stat. Neth	2006	0-89.7%	15.8	12.3
Neighbourhood social capital*	WoON	2006	−2.8-2.0	−0.22	0.83

*Based on Mohnen et al. [7]. ^a^Stat. Neth = Statistics Netherlands.

### Analytic strategy

The data were analysed by performing multilevel linear regression analyses, using Stata and MLwiN 2.24. We ran MLwiN through STATA using the ‘runmlwin’ STATA command. A three-level model (neighbourhoods, individuals, measurements) was used. This accounts for the nesting of measurements of self-rated health within individuals and individuals within neighbourhoods. We assume that the random effects are normally distributed. We specified a full variance/covariance structure between measurement occasions at level 2 in order to account for the correlation of measurements within individuals. The correlation of measurements indicates the dependency between the measurements of self-rated health. We assume there are differences between individuals in the effect of time on self-rated health; the variances and covariances of the outcome variable are variable over time and dependent on position and spacing, because the correlation is larger between nearby measurements of health than between measurements are further apart (measurements that are one year apart are more similar than measurements of health that are further apart) and the effect of time might differ between individuals with different health conditions, some people experience a more stable health or faster deterioration than others. Snijders & Bosker [51] call this a ‘fully multivariate model’ where the measurements for individuals are random at level two. This model does not have a random part at level one [51]. A description of the multilevel model used in this study can be found in Additional file 1.

We estimated an empty model (Model 0) to establish the clustering of health changes in a neighbourhood by controlling for baseline health in 2005 and differences caused by the time of measurement. Models 1 to 3 are estimated to test the first hypothesis: *A higher level of individual or neighbourhood social capital exerts a positive influence on changes in the self-rated health of people with a medically diagnosed somatic chronic disease.*

Model 1 includes variables on the individual level (including individual social capital). Model 2 adds neighbourhood social capital. Model 3 adds the neighbourhood control variables: the urbanity of the municipality and the percentage of people in the highest income quintile. In Model 4, the interaction between neighbourhood social capital and individual social capital is added to test the second hypothesis: *There is an interaction between the effect of neighbourhood social capital and individual social capital on changes in self-rated health.* Because there are only small changes in coefficients (and no significant changes) between the four different models, only the first model (the empty model) and the fifth model (the full model) are reported.

## Results

Table 3 includes correlations between individual level variables. The presence and severity of a disability correlates most highly with self-rated health.

**Table 3:** Correlation between individual characteristics, self-rated health at baseline and individual social capital at baseline (at baseline:2005)

**N**_**j**_ **= 1048**	**1**	**2**	**3**	**4**	**5**	**6**	**7**	**8**	**9**	**10**
1. Health in 2005	1.00	--	--	--	--	--	--	--		
2. Gender^a^ 0 = male	−0.037*	1.00	--	--	--	--	--	--		
3. Age	−0.107***	−0.138***	1.00	--	--	--	--	--		
4. Education level	0.121***	−0.096***	−0.321***	1.00	--	--	--	--		
5. Income	0.116***	−0.070 **	−0.082***	0.385***	1.00	--	--	--		
6. Severity of disability	−0.455***	0.092***	0.290***	−0.201***	−0.150***	1.00	--	--		
7. Number of chronic diseases	−0.169***	0.030	0.176***	−0.067***	−0.232***	0.157***	1.00	--		
8. Individual social capital	0.079***	−0.003	0.071***	−0.036*	−0.014	−0.085***	−0.013	1.00		
9. Employment status^a^ (0 = unemployed)	0.210***	−0.026	−0.661***	0.285***	0. 165***	−0.333***	−0.152***	0.014	1.00	
10. Marital status^a^ (0 = unmarried)	0.033***	−0.162***	0.035	−0.064***	−0.0618***	−0.0723***	−0.057**	0.135***	0.028	1.00

N_j_ = Number of individuals, *p ≤ 0.05, **p ≤ 0.01, ***p ≤ 0.001, ^a^dichotomous variable.

Table 4 shows correlations between neighbourhood level variables. The percentage of immigrants within a neighbourhood correlates highly with the urbanity of the area (>0.8); in more rural areas the percentage of immigrants decreases strongly. As mentioned previously, the percentage of immigrants is therefore not included in the analysis.

**Table 4:** Correlation between neighbourhood characteristics and neighbourhood social capital

**N**_**k**_ **= 259**	**1**	**2**	**3**	**4**
1. Neighbourhood social capital	1.00	--	--	
2. Urbanity of municipality	−0.512***	1.00	--	
3. Highest income quintile	−0.108***	0.024	1.00	
4. Percentage of immigrants	−0.597***	0.816***	0.0530**	1.00

N_k_ = Number of neighbourhoods, *p ≤ 0.05, **p ≤ 0.01, ***p ≤ 0.001.

The study sample consists of 1048 individuals within 259 postal code areas. The average score of self-rated health in 2005 was 53.93 and 53.03 in 2008. Twenty-nine percent of the respondents experienced a deterioration in health between the first year and last year of participation, 43% of the respondents did not experience any health changes and 27% of the respondents reported an improvement in self-rated health. A change in health of half a standard deviation or less (7 points on a scale of 0–100) was considered stable health [52].

There are only slight differences between neighbourhoods with regard to health changes of their chronically ill population. In the empty model (that includes self-rated health at baseline, see Table 5), neighbourhood level variance of health changes was not significant (2.23, se = 2.29).

**Table 5:** Multilevel regression models of individual and neighbourhood social capital on self-rated health in 2006, 2007, 2008 (Coefficients, 95% confidence intervals in parentheses)

		**Empty model (model 0)**	**Full model**
Intercept (se)		53.16 (0.54)***	44.61 (2.90)***
*Measurement level*			
Time of measurement (ref = 2008)	2006	0.21 (0.83,1.25)	0.18 (−0.86,1.22)
	2007	1.19 (0.23,2.16)*	1.17 (0.20,2.15)***
*Individual level*			
Health in 2005		0.77 (0.74,0.81)***	0.70 (0.66,0.74)***
Gender	Women		−0.26 (−1.79,1.26)
Age in 2005			−0.06 (−0.13,0.01)
Education (Ref = High)	Low		−0.08 (−2.16,1.99)
	Average		−0.65 (−2.87/1.56)
Income (Ref = More than 1600)	Less than 900		−3.85 (−6.71/-0.99)**
	900-1200		−0.45 (−2.92,2.02)
	1200-1600		−1.10 (−3.50,1.31)
	Unknown		−2.12 (−4.88,0.63)
Marital state	Married		1.56 (−0.03,3.16)*
Employment status	Employed		1.30 (−0.67,3.27)
Severity of disability (Ref = severe)	No or mild disability		6.82 (3.76,9.89)***
	Moderate disability		2.70 (−0.35,5.75)
Number of chronic diseases (Ref = three or more)	One disease		1.19 (−1.56,3.94)
	Two diseases		1.02 (−1.96,3.99)
Individual social capital in 2005			0.14 (0.04,0.25)**
Index disease (ref = cardiovascular disease)	Cancer		−0.30 (−5.05,4.45)
	Respiratory disease		0.84 (−2.08,3.76)
	Diabetes		1.11 (−1.94,4.16)
	Musculo-skeletal disease		2.06 (−1.18,5.30)
	Neurological disease		0.91 (−2.89,4.71)
	Digestive disease		0.59 (−3.95,5.14)
	Unspecified other disease		3.60 (0.58,6.63)*
Interaction neighbourhood social capital *individual social capital			0.01 (−0.11,0.13)
*Neighbourhood level*			
Neighbourhood social capital in 2006			1.03 (0.00,2.06)*
Highest income quintile			−0.12 (−0.27,0.02)
Urbanity of municipality			0.15 (−0.50,0.80)
*Measurements variance and covariance*			
Variance neighbourhood level (se)		2.23 (2.29)	0 (0)
Variance 2006 (se)		186.31 (8.72)	179.52 (8.23)
Variance 2007 (se)		205.65 (9.99)	190.74 (9.12)
Variance 2008 (se)		206.07 (11.03)	194.97 (10.32)
Covariance (2006, 2007)		94.08 (7.56)	83.75 (6.91)
Covariance (2006, 2008)		91.48 (8.01)	82.86 (7.45)
Covariance (2007, 2008)		116.33 (8.65)	103.37 (7.88)
*Intraclass correlation*			
Intraclass correlation (%)^a^		0.6	0
*Correlations between measurements*			
Correlation (2006, 2007)		0.48 (0.03)	0.45 (0.03)
Correlation (2006, 2008)		0.47 (0.03)	0.44 (0.03)
Correlation (2007, 2008)		0.57 (0.03)	0.54 (0.03)

*p ≤ 0.05, **p ≤ 0.01, ***p ≤ 0.001.

^a^Intraclass correlation = neighbourhood variation/(neighbourhood variation + mean individual variation of the three measurement moments). Mean individual variation = (variance + 2*covariance)/3.

The full model (Table 5) shows that individual social capital is significantly associated with changes in respondents’ self-rated health. Greater individual social capital at baseline is positively related to better self-rated health in later years. The same holds true for being married. Having a low income (a net equivalent income of less than 900 euros a month) as opposed to having a high income (a net equivalent income of more than 1600 euros a month) is negatively related to self-rated health. Having severe disabilities as opposed to light or moderate disabilities is also negatively related to self-rated health. Neighbourhood level variance is reduced to half the variance of the empty model when individual variables are added to Model 1 (not shown in table). This indicates that composition effects play a role in the differences in health between neighbourhoods. Variances of the different measurements of health and the covariance between them are also reduced slightly (compared to the empty model) when individual variables are added to Model 1 (not shown in table).

Beyond individual characteristics (such as individual social capital, income, marital status, and disease characteristics), a higher level of neighbourhood social capital at baseline positively relates to changes in individual self-rated health. When social capital is added in Model 2, remaining variance on the neighbourhood level is reduced to zero (not shown in table). The effect of neighbourhood social capital persists in the presence of other neighbourhood level variables. The percentage of people in the highest income quintile in a neighbourhood and the level of urbanity are not significantly related to changes in self-rated health of people with chronic illness. There is no significant interaction between neighbourhood social capital and individual social capital. Neighbourhood social capital and individual level social capital both independently impact changes in individual self-rated health.

## Discussion

Consistent with our first hypothesis we found that individual as well as neighbourhood social capital at baseline exert a significant positive effect on changes in the self-rated health of people with chronic illness. This means that people with chronic illness are less likely to experience deteriorating health when they possess higher levels of individual social capital at baseline or live in a neighbourhood with greater social capital. This finding is consistent with cross-sectional studies in the general population where beneficial effects of individual and neighbourhood social capital on health were found [7,9-15,32-34]. Our study adds two elements: First, our study population consists of people with a medically diagnosed chronic disease. The residential neighbourhood might be especially important for people with a chronic illness, as fewer of them have a paid job [53] and might be more dependent on their immediate living environment as a consequence of disabilities. Second, our study has a prospective design relating baseline social capital to changes in health.

Although neighbourhood social capital influences changes in the health of people with a chronic illness, the major part of the variation in health changes is located at the individual level and is explained by individual factors. Our study sample is too small to be able to estimate significant neighbourhood variation. In general, the literature shows that neighbourhood variation in health and effects of neighbourhood characteristics are relatively small [10,12,54,55]. More substantively, next to the neighbourhood, other sources of social capital, such as the family or workplace, might also be responsible for variations in health [56,57]. The effect of neighbourhood social capital might also differ according to the level of one’s exposure to it [7]. In order to shed more light on the influence of social capital on health it is therefore relevant to study how different sources of social capital influence people who are variably exposed to those contexts.

Inconsistent with our second hypothesis we found that the effect of neighbourhood social capital does not interact with the amount of individual social capital people possess. This suggests that social capital in the neighbourhood influences people’s self-rated health, independent of their own relations with neighbours and their own perception of the neighbourhood. The advantage of looking at individual social capital is that it is not necessarily confined to relationships within an administrative neighbourhood; it does more justice to the individual perception of what people consider to be their neighbourhood. Two fifths of the people with chronic illness in our sample report a relatively stable health. Adapting to the challenges brought about by a chronic illness might also involve the changing of internal standards about what is considered ‘good health’. It is possible that a response shift occurs because people maintain a fairly stable conception of their health over time [58]. In a sample of nonagenarians comparable rates of change of self-rated health were found [59]. Self-rated health in this sample turned out to be sensitive to changes in the number of chronic conditions and functioning [59]; however, we cannot exclude the possibility that social capital affects people’s adaptation of their perception of health rather than their actual health status.

### Strengths and limitations

We cannot control for residential relocation of the respondents in the years after baseline measurement of self-rated health and social capital because previous address in case of relocation is not recorded. On average, 10% of the Dutch population moves each year. In 2006, the majority of people who moved, moved within their own municipality (60%), which could also be within their own neighbourhood [42]. The average age of our sample was 56. Among the general population, people over 50 move significantly less (6% per year) than the average within the general population. Therefore, residential relocation might have influenced our results, but not in a strong way.

We lack data of those whose health so strongly deteriorated that they were unable to participate in future waves of the panel and those who were admitted to a nursing home. Between 2005 and 2008 approximately 15% of the attrition rate within the NPCD was caused by inability to further participate due to strongly deteriorating health, admission to a nursing home, or death. In our sample the reason of attrition (due to a strongly deteriorating health, admission to a nursing home, death or otherwise) was not related to baseline neighbourhood social capital. From the point of view of methodological bias, this is reassuring; however, it begs the question of the importance of social capital for other health related outcomes, such as the ability to live independently and mortality.

We also lack information about the panel members’ health in the years before entering the panel. Time since diagnosis varies [46] because the aim of NPCD is to provide information about a representative cross-section of Dutch people with a chronic illness. We therefore have no insight into the progression of their health (and the level of social capital) before 2005. Studying the effect of social capital on health from the onset of the disease onwards or from the moment people become residents of a neighbourhood might clarify the patterns of health development over time.

The most important strength of this study lies in its prospective design. The cross-sectional nature of previous studies renders it difficult to draw causal inferences and evidence regarding the connection between neighbourhood social capital and the progression of health has to date been lacking. This study used a representative sample of people medically diagnosed with a somatic chronic disease. It is expected that their condition causes individuals with chronic illness to experience more pronounced health changes in shorter periods of time than people from the general population, which enhanced our ability to detect the influence of social capital on health over time. This study provides evidence that the residential environment contributes to changes in the self-rated health of people with a chronic disease.

The assessment of neighbourhood social capital was derived from a representative sample of residents from neighbourhoods and not from the sample of individuals with chronic illness participating in this study. This approach enables a clear distinction between the assessment of individual social capital and neighbourhood social capital and provides an objective measure of the neighbourhood social context. The fact that neighbourhood social capital was derived from a different dataset and was constructed through ecometric analysis means that we cannot directly compare the coefficients of individual level social capital and neighbourhood social capital. This limits a causal interpretation of the relationship between individual social capital and neighbourhood social capital.

## Conclusion

Besides individual social capital, social capital on the neighbourhood level is a possible relevant influence on the health of people with chronically illness; however, there is little clarity on the mechanisms behind this relationship and the importance of the neighbourhood with regard to the promotion of health for people with chronic illness. Future studies should focus on the mechanisms underlying the relation between health and social capital, on the individual as well as the neighbourhood level, as well as focusing on the differences in this respect between the general population and people with a chronic illness. The nature of pathways as well as their importance might differ between both population groups. Social support might be very important for people with a chronic illness. Influenced by norms that are operative within the neighbourhood, people might feel more motivated to perform healthy behaviours. In the case of physical activity e.g., this might be less intensive than in the general population, but still preventative of further deterioration of health. Contacts in the neighbourhood can facilitate the diffusion of health-related norms and information [60,61]. A higher level of social capital might also create an impetus to undertake collective action on the basis of mutual trust and willingness to intervene for the common good. Through higher levels of social capital, residents might be able to lobby for better services and health-promoting amenities in the neighbourhood. Furthermore, social capital can influence mental health through a positive atmosphere of mutual recognition and respect [60].

Current policy of the Dutch government in the area of support and long-term care is to devolve responsibility to municipalities and to people themselves and their social networks. People with support needs will be stimulated to utilize their social network and neighbourhood resources before appealing to municipal services [62]. An important assumption in this policy is that people indeed possess a social network and live in neighbourhoods that are able and willing to provide needed support. Another assumption is that this policy does not lead to increasing inequalities between people with differing levels of access to support. Studies on the working mechanisms of individual and neighbourhood social capital play an important role in this [63]. While assessing these policy changes, it is important to study a broader range of effects, e.g. whether people are able to live independently for a longer time in communities with more social capital. Furthermore, it is also important to not only examine changes in health but also changes in social capital in order to discover how developments in the social environment influence health over time. Investigating the effect of neighbourhood social capital on the progression of health, and the mechanisms underlying this effect, provides a useful framework with which to identify what constitutes a health-supporting living environment [49].

## Competing interests

The authors declare that they have no competing interests.

## Authors’ contributions

GW drafted the manuscript and performed the statistical analysis. MKW conceived of the study, provided background information for this manuscript, and did provisional analysis. SM advised about ecometrics and provided data for this manuscript. MR has developed the NPCD, advised about the use of the data from the NPCD, and helped to draft the manuscript. PS helped perform the statistical analysis. PG conceived of the study, participated in its design and coordination, and helped draft the manuscript. All authors read and approved the final manuscript.

## Pre-publication history

The pre-publication history for this paper can be accessed here:

http://www.biomedcentral.com/1471-2458/14/675/prepub

## Supplementary Material

Additional file 1 Description of the multilevel repeated measures used in this study. Click here for file
