# Supplementary material for: A prospective analysis of the effect of neighbourhood and individual social capital on changes in self-rated health of people with chronic illness
Source: BMC Public Health. 2014 Jul 3;14:675. doi: 10.1186/1471-2458-14-675 (PMC4105130; doi:10.1186/1471-2458-14-675)
Supplement: Additional file 1 — Description of the multilevel repeated measures used in this study. [file 1471-2458-14-675-S1.docx]

Additional file 1 - Description of the multilevel repeated measures used in this study

$$Y_{tij}=\beta_{0}+\beta_{t}x_{t}+\beta_{i}x_{0ij}+\beta_{j}z_{j}+v_{j}+U_{ti}$$

t = time 2006 .. 2008

i = individual 1 ..1048

j = neighbourhood 1 .. 259

$Y_{tij}$ outcome score at moment (t) for an individual (i) in neighbourhood (j)

$\beta_{0}$ intercept

$\beta_{t}x_{t}$ deviation ($\beta_{t}$) from intercept for time ($x_{t}$)

$\beta_{i}x_{0ij}$ effect for variables measured at the individual level that are constant over time

$\beta_{j}z_{j}$ effect for neighbourhood variables that are constant over time

$v_{j}$ random effect, the between neighbourhood variance

$U_{ti}$ random effect, complete time dependent between and within individual

variance/covariance matrix $\left( \begin{matrix} \sigma_{2006} & \sigma_{2006,2007} & \sigma_{2006,2008} \\ \sigma_{2006,2007} & \sigma_{2007} & \sigma_{2007,2008} \\ \sigma_{2006,2008} & \sigma_{2007,2008} & \sigma_{2008} \end{matrix} \right)$

on the diagonal we have the within individual yearly (error) variance
and off the diagonal the between individual covariance between two years.
All parameters of this matrix are estimated from the data and are unstructured, meaning they can all be different. All the random effects are assumed to be normally distributed.
Note that this variance/covariance matrix is modelled at the level of the individuals, the lowest level, the individual measurements, is empty.
